# Bacterial vaginosis and human immunodeficiency virus infection

**DOI:** 10.1186/1742-6405-4-25

**Published:** 2007-10-22

**Authors:** Gregory T Spear, Elizabeth St John, M Reza Zariffard

**Affiliations:** 1 Department of Immunology/Microbiology, Rush University Medical Center, Chicago, IL 60612 USA

## Abstract

Epidemiologic studies indicate that bacterial vaginosis (BV), a common alteration of lower genital tract flora in women, is associated with increased susceptibility to HIV infection. Other recent studies show that HIV is detected more frequently and at higher levels in the lower genital tract of HIV-seropositive women with BV. In vitro studies show that genital tract secretions from women with BV or flora associated with BV induce HIV expression in infected cells. The increased HIV expression appears to be due at least in part to activation through Toll-like receptors (TLR), specifically TLR2. Further research is needed to elucidate how BV contributes to HIV acquisition and transmission.

## Review

### Bacterial vaginosis

Bacteria colonize the lower genital tract of most women and the predominant species of bacteria in healthy women is lactobacilli. Commonly found vaginal lactobacillus species include *Lactobacillus crispatus*, *L. gasseri*, *L. jensenii *and *L. iners *[1,2]. Bacterial vaginosis (BV) is characterized by an alteration of genital tract flora such that the predominant bacteria are no longer lactobacilli, but instead consist of polymicrobial communities of multiple genera of gram positive and gram negative organisms [3]. *Gardnerella vaginalis*, *Prevotella *sp.,*Bacteroides *sp., *Peptostreptococcus *sp., *Mycoplasma hominis *and *Mobiluncus *sp. as well as other recently described bacteria are commonly found in BV [2,3]. Lactobacilli, usually *L. iners*, are also frequently present in BV, but make up a relatively small proportion of the total flora [2,4]. BV has been noted to be the most prevalent vaginal disorder in adult women worldwide with the frequency depending on the group that is studied [5]. BV is found in 24% to 37% of women attending STD clinics but seen at lower rates in women that are not sexually active.

BV is associated with an increased risk of infections by HIV and some other organisms as discussed below, as well as with increased risk of preterm birth, which is a leading cause of infant death in the United States [6-8]. Treatment of BV can reduce preterm birth in high risk cases [7,9]. BV is also associated with miscarriage and pelvic inflammatory disease [10-12].

Diagnosis of BV is commonly made by examination of four criteria: vaginal fluid pH (BV results in a pH >4.5); presence of clue cells (bacteria-coated epithelial cells); a homogenous discharge; and production of an amine odor when KOH is added to vaginal fluid [13]. Gram stains of vaginal fluid can also aid in diagnosis of BV [14].

Oral or intravaginal antibiotic treatment with metronidazole or clindamycin cures BV in most women, but BV can resolve spontaneously in nearly a third of subjects [15-18]. However, BV recurs in a significant fraction of treated women.

### In vivo studies of the effects of BV on HIV susceptibility and expression

Several cross-sectional studies performed in Thailand [19], Uganda [20], and Malawi [21,22] showed that women with BV had an increased incidence of HIV infection. While suggestive, these studies do not prove a cause and effect relationship between BV and HIV infection. However, a prospective study in Kenya [23] showed that the presence of BV and the absence of lactobacilli or absence of hydrogen peroxide-producing lactobacilli upon examination were all significantly associated with acquisition of HIV infection at follow-up.

There is also evidence, some of it through cross-sectional studies, that the presence of BV increases the risk of infection with several other sexually transmitted infections (STI), including herpes simplex virus type 2 (HSV-2), gonorrhea, *Trichomonas vaginalis *and *Chlamydia trachomatis *[23-25]. All of these STI have been suggested to increase susceptibility of women to sexual transmission of HIV [26], and so BV may both directly increase HIV susceptibility and indirectly increase it by increasing the number of women with these other STI.

While the above studies suggest that BV can influence susceptibility of women to HIV infection, other recent studies suggest that BV increases expression of HIV in the lower genital tract of women that are already infected with HIV. Thus, the levels of HIV, as assessed by HIV RNA, and the detection frequency of HIV in the genital tract are significantly higher in the genital tract of women with BV when compared to women without BV [27,28]. HIV levels were inversely correlated with levels of lactobacilli but positively correlated with *Mycoplasma hominis *[27]. An additional study showed that women with lower levels of vaginal lactobacilli had higher genital tract HIV [29].

A number of mechanisms have been suggested that could account for the increase in susceptibility to HIV and/or increased expression of HIV in the genital tract (Table 1) [26,30]. These include decreased levels of hydrogen peroxide-producing lactobacilli, production by BV flora of enzymes (e.g. mucinases) that degrade protective mechanisms such as mucous, or production by BV flora of stimulatory substances that increase influx of target cells, HIV expression or infection of cells (see below). In fact, BV is associated with increased levels of pro-inflammatory cytokines such as IL-1β and IL-8 [Reviewed in [31]]. IL-1β can induce the production of other pro-inflammatory cytokines, and IL-8 is known to recruit immune cells, thus possibly increasing the number of cellular targets for HIV infection [32].

**Table 1:** Possible mechanisms of bacterial vaginosis effects on HIV transmission and HIV replication

Increased vaginal pH
Decreased levels of hydrogen peroxide-producing lactobacilli
Production by BV flora of enzymes or substances that inhibit anti-HIV immunity
Increased influx of cells susceptible to HIV infection
Increased HIV expression and/or infection

### In vitro studies of the relationship between BV and HIV

A number of in vitro studies show that genital tract fluids from women with BV are highly stimulatory for immune cells and can up-regulate expression of HIV. Thus, incubation of the chronically-HIV-infected monocytic cell line U1 with genital fluid from women with BV substantially increased HIV expression [33-36]. In contrast, genital fluid collected from women without BV did not induce HIV expression. HIV expression was also induced in T cell lines and in peripheral blood mononuclear cells by genital fluids from women with BV [35,37]. The substances in genital fluids that stimulated HIV expression in cells were found to function through activation of NF-kB [37].

Bacteria from BV have also been tested for their ability to stimulate HIV expression in cells. *Gardnerella vaginalis*, the bacterium most frequently isolated in BV, significantly induced HIV expression in U1 cells [38,39]. Lysozyme treatment reduced U1 activation suggesting a cell wall component of *G. vaginalis *was involved in stimulation of the U1 cells. Anaerobes *Peptostreptococcus asaccharolyticus *and *Prevotella bivia *also stimulated HIV expression [40] as did non-anaerobic bacteria *Mycoplasma hominis *and *Streptococcus *[39]. In contrast, other bacteria found in genital samples including *Bacteroides ureolyticus*, *Peptostreptococcus anaerobius*, and *Lactobacillus acidophilus *did not stimulate HIV expression [40].

Taken together, many of the above studies suggested that the HIV-stimulatory activity in genital fluids acted through Toll-like receptors (TLR). For example, genital fluids stimulated HIV expression through the NF-kB pathway [41], and stimulation of cells through TLR is well documented to activate NF-kB [42]. Also, many of the ligands for TLR are bacterial products [42] and BV mucosal fluids would be expected to contain such products. A recent study using the 293 cell line modified to express either TLR2, TLR4 or control cells expressing no functional TLR, directly determined whether mucosal fluids from women with BV stimulated cells through TLR [41]. The results showed that genital fluids from women with BV stimulated cells predominantly through TLR2, while surprisingly there was relatively little stimulation through TLR4. In contrast, fluids from women without BV stimulated cells relatively little through either TLR2 or TLR4. Further, the TLR2-positive cells supported higher levels of expression of the HIV promoter when exposed to genital secretions from women with BV, suggesting that HIV-infected cells in the genital tract might express higher levels of HIV during episodes of BV. Other studies showed that genital tract fluid from women with BV can stimulate lymphocytes and other cells to express higher levels of TLR4 and TNF-α [43].

**Figure 1:**
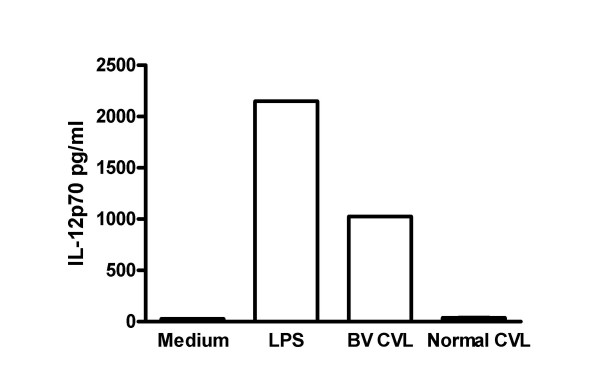
Bacterial Vaginosis induces IL-12p70 production by Dendritic Cells. Monocyte-derived dendritic cells (MDDC) were produced from monocytes isolated from the blood of normal donors using standard methods [46]. MDDC were incubated for 48 hours with either culture medium alone (Medium), lipopolysaccharide at 1 μg/ml (LPS), or genital tract secretions collected by cervicalvaginal lavage from women with BV (BV CVL) or normal flora (Normal CVL). The BV CVL and Normal CVL were pools of equal amounts of CVL from 15 and 14 women respectively. Status of CVL donors was determined by gram stain. Supernatants were harvested and analyzed for IL-12p70 by ELISA.

Dendritic cells (DC), cells important for antigen processing and presentation to the immune system, are found in the lower genital tract and are known to express both TLR2 and TLR4. DC are suggested to be one of the first cells that take up HIV during sexual transmission [44,45]. DC are also potent antigen presenting cells whose function would be important for vaccination against a number of mucosal pathogens, including HIV. We have investigated the hypothesis that genital tract secretions from women with BV might substantially affect either DC antigen presenting function or DC uptake and infection by HIV. We observed that secretions from women with BV potently stimulate secretion of IL-12 by monocyte-derived dendritic (MDDC) (Figure 1) [46]. Genital fluid from women with BV also increased MDDC secretion of IL-23 and p40 and upregulated cell surface HLA-DR, CD40 and CD83. Further, BV fluids decreased MDDC endocytic ability (a marker of stimulation and maturation of DC) and increased proliferation of T cells in an allogeneic MLR with MDDC as the antigen presenting cells [46]. Genital fluids from women without BV had much lower or no stimulatory activity for MDDC. These studies suggest that BV may substantially affect local DC antigen presenting function in women.

**Figure 2:**
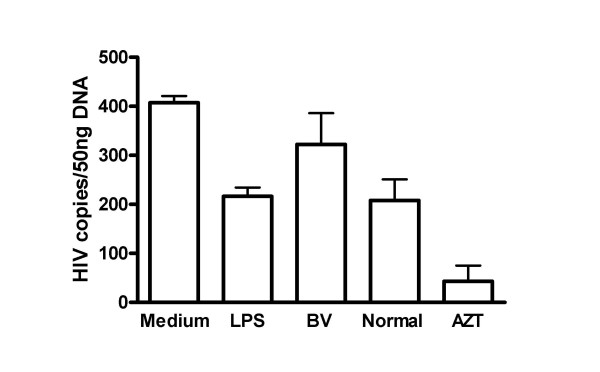
Effect of Bacterial Vaginosis on HIV infection of MDDC. MDDC were produced and treated with either Medium alone, BV CVL, Normal CVL or LPS for 48 hr as described in the Figure 1 legend. Treated MDDC were incubated with HIV-1_Bal _for 24 hr. DNA was then isolated from the MDDC and analyzed for HIV DNA copies by real time PCR. Bars represent mean + standard error.

**Figure 3:**
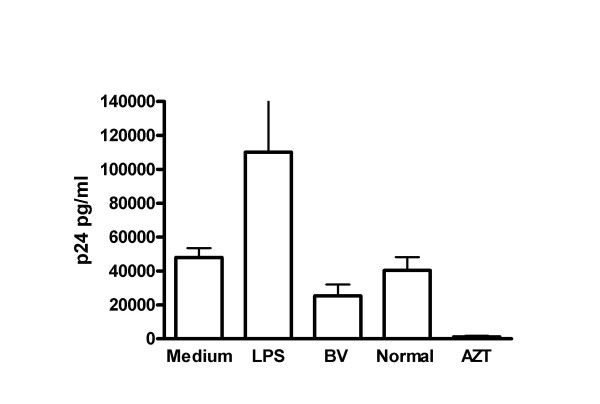
Effect of Bacterial Vaginosis on HIV transfer from MDDC to T cells. MDDC were produced and treated as described in the Figure 2 legend and then exposed to HIV-_Bal _for 2 hours. Free virus was removed by washing and MDDC were incubated with PHA stimulated PBMC. Five days later supernatants were harvested analyzed for p24 production by ELISA. Bars represent mean + standard error.

Since the above studies showed that BV genital secretions potently stimulate DC, we hypothesized that this stimulation might increase infection of DC or enhance the ability of DC to transfer HIV to T cells. However, our studies to date do not show BV enhancing HIV infection of DC (Fig. 2) or transfer of HIV by DC to T cells (Fig. 3). In fact, BV genital secretions appear to suppress HIV transfer to T cells (Fig. 3). While our studies currently do not support a role for direct effects of BV genital secretions on DC in enhancing HIV transmission, these findings do not rule out the possibility that BV promotes HIV transmission by altering DC function or trafficking in vivo.

## Conclusion

While it has become evident that BV has effects on HIV transmission, HIV genital tract levels and HIV expression in vitro, further work is needed to identify the mechanisms responsible for these effects. For example, questions remain regarding the direct contribution of bacterial flora versus indirect mechanisms through immune cells, immune mediators such as cytokines or other mediators. New in vitro experimental systems or animal models are needed to help elucidate these mechanisms and are likely to lead to increased understanding of ways to prevent the spread of the HIV epidemic.
